# Development and validation of a Diabetes Risk Score for screening undiagnosed diabetes in Sri Lanka (SLDRISK)

**DOI:** 10.1186/s12902-016-0124-8

**Published:** 2016-07-25

**Authors:** P. Katulanda, N. R. Hill, I. Stratton, R. Sheriff, S. D. N. De Silva, D. R. Matthews

**Affiliations:** 1 Oxford Centre for Diabetes, Endocrinology and Metabolism, University of Oxford, Oxford, UK; 2 Diabetes Research Unit, Department of Clinical Medicine, Faculty of Medicine, University of Colombo, Colombo 08, Sri Lanka; 3 Nuffield Department of Primary Care Health Sciences, University of Oxford, Oxford, UK; 4 Gloucestershire Hospitals NHS Foundation Trust, Gloucester, UK

**Keywords:** Diabetes, Screening, Risk score, Sri Lanka, South Asian

## Abstract

**Background:**

Screening for undiagnosed diabetes is not widely undertaken due to the high costs and invasiveness of blood sampling. Simple non-invasive tools to identify high risk individuals can facilitate screening. The main objectives of this study are to develop and validate a risk score for screening undiagnosed diabetes among Sri Lankan adults and to compare its performance with the Cambridge Risk Score (CRS), the Indian Diabetes Risk Score (IDRS) and three other Asian risk scores.

**Methods:**

Data were available from a representative sample of 4276 adults without diagnosed diabetes. In a jack-knife approach two thirds of the sample was used for the development of the risk score and the remainder for the validation. Age, waist circumference, BMI, hypertension, balanitis or vulvitis, family history of diabetes, gestational diabetes, physical activity and osmotic symptoms were significantly associated with undiagnosed diabetes (age most to osmotic symptoms least). Individual scores were generated for these factors using the beta coefficient values obtained in multiple logistic regression. A cut-off value of sum = 31 was determined by ROC curve analysis.

**Results:**

The area under the ROC curve of the risk score for prevalent diabetes was 0.78 (CI 0.73–0.82). In the sample 36.3 % were above the cut-off of 31. A risk score above 31 gave a sensitivity, specificity, positive predictive value and negative predictive value of 77.9, 65.6, 9.4 and 98.3 % respectively. For Sri Lankans the AUC for the CRS and IDRS were 0.72 and 0.66 repectively.

**Conclusions:**

This simple non-invasive screening tool can identify 80 % of undiagnosed diabetes by selecting 40 % of Sri Lankan adults for confirmatory blood investigations.

## Background

Diabetes mellitus is increasing worldwide [1]. Type 2 diabetes accounts for over 90 % of prevalent cases in high risk populations such as South Asians and Pima Indians [2, 3]. Among those with type 2 diabetes 30 to 76 % remain undiagnosed according to different estimates [2, 4–7]. In a national level epidemiological study conducted in 2006 in Sri Lanka, the diabetes prevalence was reported as 10.3 % with 36 % remaining undiagnosed [2]. Early diagnosis is important as in the United Kingdom Prospective Diabetes Study (UKPDS), 37 % of those newly diagnosed with type 2 diabetes had retinopathy [8]. A cross-sectional study in Sri Lanka reported 15 % retinopathy and 25 % neuropathy in newly diagnosed diabetic subjects [9]. These data suggest that type 2 diabetes may remain undiagnosed for many years leading to adverse diabetes related outcomes.

Early diagnosis and optimisation of therapy may improve outcomes in patients with diabetes, as tight control of blood glucose and blood pressure has been shown to reduce the incidence of microvascular complications in type 2 diabetic subjects [10, 11] and control of lipids reduces macrovascular disease and mortality in people with diabetes [12]. This reduction of risk of microvascular complications, myocardial infarcton and death from any cause persisted in 10 years of post-trial followup [13]. Since the diagnosis of diabetes requires invasive blood sampling and laboratory investigations, there is an ongoing debate about the benefit and the potential harm of screening for diabetes [14–17]. A recent study from UK has reported limited psychological impact on patients from screening for diabetes [18]. We hypothesise that if sensitive, specific and low cost tools (that are non-invasive) can be developed, large scale community level diabetes screening can be undertaken with a potential for the improvement of outcomes in otherwise undiagnosed people.

Questionnaire based risk screening tools have been developed for the detection of diabetes in other populations [16, 19–21]. The aim of this study was to develop and validate a screening tool for undiagnosed diabetes in Sri Lanka based on demographic information. The performance of the risk tools developed for White Europeans in the UK by Griifin et al. [16] and Asian Indians by Ramachandran et al. [22] were compared with our risk score.

## Methods

Data from 4276 subjects without previously diagnosed diabetes from the Sri Lanka Diabetes and Cardiovascular Study (SLDCS) were used for risk score development and validation. SLDCS was a nationally representative study conducted in seven out of the nine provinces in Sri Lanka using a probability cluster-sampling technique. The study was approved by the Ethical Review Committee of the Faculty of Medicine – University of Colombo. Informed written consent was obtained from all participants. The sampling and data collection of this study has been reported previously [2]. Two thirds of this study population were randomly selected for the development of the risk score. The remaining third of the subjects were used for validation.

We used methods adopted for the development of the Finnish and Cambridge Risk scores [16, 21, 22]. We performed univariate regression analysis to select variables that were associated with undiagnosed diabetes. Variables that could be easily measured in the community with minimal expertise and resources were included. We did not include area of residence, occupation and income. Multiple logistic regression analysis was carried out with newly diagnosed diabetes as the dependent variable and those variables identified from the univariate analyses at significance level <0.05.

The β coefficient values obtained in the logistic regression analysis (without stepwise elimination) were used to derive a risk score. The sum of β coefficients in multiple regression analysis can be used to derive cumulative regression coefficients [23]. Data were analysed using SPSS version 14 (SPSS Inc, Chicago IL, USA). Similar methods have been adopted in the development of the Finnish and Cambridge Risk scores [16, 21, 22]. The score was based on the beta coefficient multiplied by a factor of ten to allow integer scores. The total score was calculated as the sum of the individual weighted scores.

Diabetes was diagnosed using the 1989 WHO criteria using fasting and 2-h OGTT plasma glucose [24]. Physical activity was recorded (all activities including occupational, daily living and leisure time) using the short format of International Physical Activity Questionnaire (IPAQ) and categorised as low, moderate and active according to the IPAQ categorisation [25]. Hypertension was diagnosed when systolic blood pressure was ≥140 mmMg/ diastolic blood pressure ≥90 mmMg or if the subjects were on antihypertensive medications [26]. Balanitis was defined as persistent itching or soreness of the glans penis and vulvitis as persistent itching, soreness and discharge from the vulva. Frequent thirst, polyuria and nocturia were considered as osmotic symptoms.

The performance of the risk score in detecting prevalent undiagnosed diabetes was evaluated using receiver-operating characteristic (ROC) curve analysis. The risk score value with combined highest sensitivity and specificity was considered the optimal cut-off value. The risk score was validated in the remaining one third of the population. The performance of the Cambridge Risk Score (CRS), the Indian Diabetes Risk Score (IDRS), two Chinese risk scores and a Thai risk score [16, 22, 27–29] when applied to the Sri Lankan population were compared with the newly derived risk score (SLDSRISK).

## Results

In the 4276 subjects 196 had newly diagnosed diabetes. Metabolic, anthropometric and lifestyle characteristics are shown in Table 1. Women had higher mean 2-h plasma glucose and BMI compared to men (*p* < 0.001). They also had higher prevalence of physical inactivity (*p* < 0.001) and infections in the genital area compared to men (*p* < 0.001). Table 2 compares development and validation cohorts which shows no significant difference in the two groups with regard to any of the parameters.

**Table 1:** Characteristics of the sample according to gender

	Male	Female	All	*p value
*n*				
Age (years)	45.5 (15.8)	45.0 (14.5)	45.2 (15.1)	0.363
FPG (mmol/l)	4.9 (1.0)	4.8 (1.2)	4.9 (1.1)	0.375
2-h PG (mmol/l)	5.7 (2.9)	6.4 (2.8)	6.1 (2.8)	< 0.001
BMI (kg/m^2^)	20.9 (3.7)	22.0 (4.5)	21.6 (4.2)	< 0.001
Waist circumference (cm)	77.4 (10.8)	76.1 (12.1)	76.6 (11.6)	< 0.001
Male sex (%)	–	–	40.0	–
Physical inactivity (%)	14.5	7.9	10.5	< 0.001
Family history of diabetes (%)	21.3	24.5	23.2	0.016
Hypertension (%)	24.3	24.5	24.4	0.955
Gestational diabetes (%)		1.2	–	–
Osmotic symptoms (%)	14.1	13.6	13.8	0.661
Balanitis or vulvitis (%)	1.1	5.4	3.7	< 0.001

*p value – between male and female

**Table 2:** Characteristics of the sample according to development and validation cohort

	Development	Validation	All	*p value
*n*				
Age (years)	45.3 (15.1)	45.1 (14.9)	45.2 (15.1)	0.743
FPG (mmol/l)	4.9 (1.1)	4.9 (1.2)	4.9 (1.1)	0.796
2-h PG (mmol/l)	6.1 (2.9)	6.2 (2.8)	6.1 (2.8)	0.796
BMI (kg/m^2^)	21.6 (4.3)	21.6 (4.2)	21.6 (4.2)	0.815
Waist circumference (cm)	76.6 (11.8)	76.6 (11.3)	76.6 (11.6)	0.994
Male sex (%)	39.6	40.5	40.0	0.560
Physical inactivity (%)	10.7	10.2	10.5	0.971
Family history of diabetes (%)	23.3	23.2	23.2	0.971
Hypertension (%)	24.6	24.1	24.4	0.706
Gestational diabetes (%)	0.7	0.8	0.7	0.979
Osmotic symptoms (%)	13.0	15.4	13.8	0.027
Balanitis or vulvitis (%)	3.6	3.9	3.7	0.677

*p value – between development and validation

### Model development

**Fig. 1:**
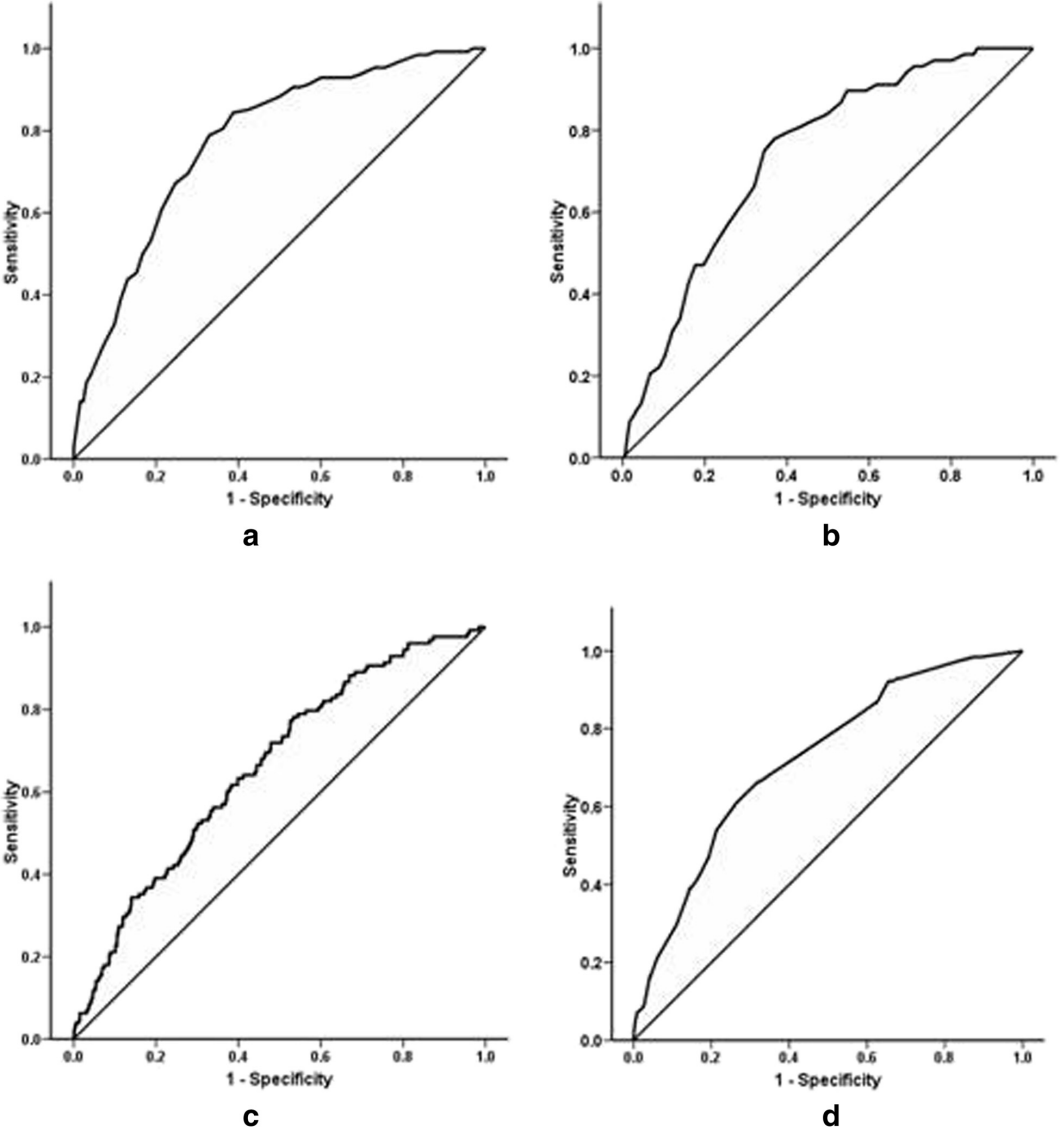
ROC curves for SLDRISK, CRS and IDRS. **a** and **b** shows SLDRISK on cohorts 1 and 2 respectively. **c** shows the CRS on cohort 1. **d** shows IDRS on cohort 1

In 2826 subjects in the development cohort 128 had newly diagnosed diabetes (46 men and 86 women). The results of the multiple logistic regression analysis (without stepwise elimination) and the individual scores are shown in Table 3. The ROC curve associated with prevalent undiagnosed diabetes in the model development is shown in Fig. 1a. The AUC of the ROC curve for detecting prevalent undiagnosed diabetes was 78 %. A cut-off value of 31 had maximal combined sensitivity and specificity. The risk score (Sri Lanka Diabetes Risk Score – SLDRISK) is available as a supplementary file.

**Table 3:** Multiple logistic regression analysis and individual scores for variables included in the risk score

Variables (*p* value for diabetes)	Category	Odds ratio (95 % CI)	β	Contribution to Risk Score (10 × β)
Age (years) (*p* = 0.000)	<30	Reference		0
	30–39	2.6 (0.9–7.8)	0.95	10
	40–49	4.8 (1.7–13.7)	1.57	16
	≥50	5.0 (1.8–14.2)	1.61	16
BMI (kg/m^2^) (*p* = 0.000)	<18.5	Reference		0
	18.5–22.9	1.7 (0.7–3.9)	0.52	5
	≥23.0	2.1 (0.8–5.2)	0.72	7
Waist circumference (cm) (*p* = 0.000)	F < 70 / M <75	Reference		0
	F (70–79)/M (75–84)	1.9 (0.9–4.1)	0.65	7
	F ≥ 80/M ≥ 85	2.9 (1.3–6.6)	1.00	10
Hypertension (*p* = 0.000)	Absent	Reference		0
	Present	2.3 (1.6–3.4)	0.84	8
Family history of diabetes (*p* = 0.000)	Present	Reference		0
	Absent	1.7 (1.1–2.5)	0.52	5
Physical activity level (*p* = 0.008)	Sufficiently active	Reference		0
	Moderately active	1.2 (0.8–1.8)	0.17	2
	Inactive	1.4 (0.8–2.4)	0.32	3
Gestational diabetes (*p* = 0.270)	Absent	Reference		0
	Present	1.4 (0.3–6.8)	0.36	4
Balanitis or vulvitis (*p* = 0.034)	Absent	Reference		0
	Present	2.0 (0.9–4.4)	0.71	7
Osmotic symptoms (*p* = 0.024)	Absent	Reference		0
	Present	1.2 (0.7–1.9)	0.14	1

### Model validation

When we applied SLDRISK to the remaining one third of the sample (cohort 1450 people, 68 with newly diagnosed Type 2 diabetes, 38.9 % had a score above 31 (risk-score positive). The risk score has a sensitivity of 77.9 % and a specificity of 63.0 %. The score has a positive predictive value (PPV) of 9.4 % and a negative predictive value (NPV) of 98.3 % for predicting prevalent diabetes in the remaining one third of the subjects (Fig. 1a). The ROC curve analysis in the validation sample showed an AUC of 0.74.

### Comparison of SLDRISK with Cambridge, Indian, Chineses and Thai Diabetes Risk Scores

The AUC of the CRS when applied to the Sri Lanka sample was 0.66 (0.62–0.71) with the cut-off for optimum combined sensitivity and specificity being 0.052 (Fig. 1c). Accordingly, the sensitivity, specificity, PPV and NPV for the CRS were 54.4, 59.3, 6.2 and 96.4 % (Table 4). The AUC of the IDRS was 0.72 (0.68–0.76) (Fig. 1d). The optimal cut-off for the IDRS was 21.5. The sensitivity, specificity, PPV and NPV of IDRS were 66.2, 66.1, 8.8 and 97.5 % respectively.

**Table 4:** The performance of SLDRISK, CRS and the IDRS in the Sri Lankan adult population

	SLDRISK	IDRS	CRS
AUC	0.78	0.72	0.66
Sensitivity (%)	77.9	66.2	54.4
Specificity (%)	65.6	66.1	59.3
Positive predictive value (%)	9.4	8.8	6.2
Negative predictive value (%)	98.3	97.5	96.4

Two Chineses risk scores and a Thai risk score were also applied to the Sri Lanka sample. The optimal cut-offs for risk scores 1 [27], 2 [28] and 3 [29] were 7.5, 27.5 and 6.5 respectively. SLDRISK performed better than all these other Asian risk scores in predicting prevalent diabetes in Sri Lankan population (Table 5).

**Table 5:** The performance of SLDRISK compared to two Chinese risk scores and a Thai risk score in the Sri Lankan adult population

	SLDRISK	RS 1 [27]	RS 2 [28]	RS 3 [29]
AUC	0.78	0.70	0.77	0.76
Sensitivity (%)	77.9	65.5	71.0	62.2
Specificity (%)	65.6	64.9	73.0	69.5
Positive predictive value (%)	9.4	7.7	11.1	8.7
Negative predictive value (%)	98.3	97.6	98.1	97.4

## Discussion

In this study we have developed and validated a tool for community level screening of undiagnosed diabetes in a high risk South Asian population. The SLDRISK can be used by local health care workers in a village setting or in health centres wihtout needing hospital or laboratory support. It can be used with minimal expertise. The AUC of 0.78 for the SLDRISK indicated satisfactory predictability of prevalent undiagnosed diabetes in Sri Lankan adults. The score would detect 78 % of those with undiagnosed diabetes in the community by identifying 39 % of risk score positive adults in the general population. This non-invasive tool has the potential to be used for targeting individuals for definitive diabetes diagnostic screening at a population level.

Many systematic reviews have been done over the years on diabetic risk scores. Collins et al. reviewed 39 similar studies done before 2011 [30]. Age, Family history of diabetes, hypertension, BMI and waist circumference were the most frequenly used risk predictors in those studies. All these risk predictors are included in our study as well.

The CRS risk score which showed an AUC of 0.80 for the White Europeans was less predictive for the Sri Lankan population (AUC 0.66). The positive predictive value of CRS for Sri Lankans at an optimal cut-off value of 0.052 was 6.2 % compared to 9.4 % in the SLDRISK. The IDRS performed comparable to the SLDRISK in Sri Lankans (AUC 0.72 vs. 0.78, PPV 8.8 vs. 9.4). This highlights the importance of using ethnic specific tools in epidemiology and disease screening. In the SLDRISK we have used common diabetes symptoms that were not used in the CRS and the IDRS to increase the sensitivity of the risk score.

Our projections of diabetes in Sri Lanka indicate a prevalence of 13.9 % in 2030 [2]. Other countries in South Asia like India, Pakistan and Bangladesh are also having high prevalence of diabetes [31–37]. This has necessitated effective primary and secondary preventive measures to prevent the rising prevalence as well as diabetes related adverse health outcomes in this ethnic group. Therefore, low cost and non-invasive epidemiological tools such as SLDRISK are of particular value in South Asian countries with limited healthcare resources and high disease burden.

## Conclusions

In conclusion, we have developed and validated a simple, low-cost and a non-invasive tool for stepwise community level screening of undiagnosed diabetes in a high risk South Asian population in Sri Lanka. Our risk score is comparable to the one developed for Asian Indians by Ramachandran et al. and can be used in Sri Lanka and perhaps among other South Asians for community level screening programmes for diabetes.

## Abbreviations

CRS, Cambridge Risk Score; IDRS, Indian Diabetes Risk Score; BMI, body mass index; ROC, receiver operating characteristic; AUC, area under the curve; SLDCS, Sri Lanka Diabetes and Cardiovascular Study; IPAQ, International Physical Activity Questionnaire; SLDRISK, Sri Lanka Diabetes Risk Score
